# B Vitamins and Their Roles in Gut Health

**DOI:** 10.3390/microorganisms10061168

**Published:** 2022-06-07

**Authors:** Khandkar Shaharina Hossain, Sathya Amarasena, Shyamchand Mayengbam

**Affiliations:** Department of Biochemistry, Memorial University of Newfoundland, St. John’s, NL A1C 5S7, Canada; kshossain@mun.ca (K.S.H.); ysaamarasena@mun.ca (S.A.)

**Keywords:** B vitamins, microbiota, gut health

## Abstract

B vitamins act as coenzymes in a myriad of cellular reactions. These include energy production, methyl donor generation, neurotransmitter synthesis, and immune functions. Due to the ubiquitous roles of these vitamins, their deficiencies significantly affect the host’s metabolism. Recently, novel roles of B vitamins in the homeostasis of gut microbial ecology and intestinal health continue to be unravelled. This review focuses on the functional roles and biosynthesis of B vitamins and how these vitamins influence the growth and proliferation of the gut microbiota. We have identified the gut bacteria that can produce vitamins, and their biosynthetic mechanisms are presented. The effects of B vitamin deficiencies on intestinal morphology, inflammation, and its effects on intestinal disorders are also discussed.

## 1. Introduction

B vitamins are a group of water-soluble organic compounds essential for several physiological functions of almost all living organisms [1]. The functional roles of these micronutrients are diverse, and they primarily act as cofactors in a plethora of enzymatic reactions. One or more B vitamins are involved in every energy-producing reaction of the cells, such as the mitochondrial citric acid cycle and cellular aerobic respiration [2]. They also play vital roles in immune functions, neurotransmitter synthesis, one-carbon metabolism, cell signalling, and even nucleic acid biosynthesis [3,4,5]. For instance, coenzyme A, an active form of vitamin B5, is a ubiquitous molecule in the cells and acts as a cofactor or direct precursor for numerous cellular intermediates [6,7].

Our gut is one of the most significant parts of the body. It acts as a way to transit and absorb what we eat [8]. It hosts different types of microorganisms such as bacteria, eukarya, and archaea. These microorganisms are commonly known as the “gut microbiota.” They comprise Bacteroidetes and Firmicutes as the dominant phyla and Actinobacteria, Proteobacteria, and Verrucomicrobia as minority members [9,10]. They maintain a symbiotic relationship with the host and protect against harmful pathogens [11]. They enhance energy utilization through intestinal fermentation [12] and regulate host immune function and signalling molecules [13]. The human gut microbiota is unique and relatively stable, and thus highly resilient to change [14]. In spite of this, they are also dynamic, and their composition is remodelled during different stages of our lifespan [15]. Several factors, such as the birth method of a child [16,17]; the age of the host [18]; lifestyle; medications; and, most importantly, diet [19,20,21], significantly modulate the composition of the gut microbiota. 

Nevertheless, our gut also harbours bacteria that produce B vitamins, including biotin, cobalamin, folate, niacin, pantothenate, pyridoxine, riboflavin, and thiamin, but in limited amounts [22]. Several gut bacteria require specific vitamins for their growth, and at the same time, auxotrophic bacteria create competition between them. Deficiencies of such vitamins impair normal cellular metabolism and instigate the development of several chronic diseases in humans. Therefore, B vitamins are essential not only for the host but also for the bacteria living in the gut. A dietary supply of these vitamins is essential to meet the host’s daily requirements. B vitamins play crucial roles in shaping the diversity and richness of the gut microbiota. Considerable evidence has demonstrated that a healthy gut lies in a healthy microbial ecology. Therefore, it is indispensable to segregate the relationship between gut microbiota and a healthy gut. This review highlights the functions and effects of each B vitamin, the bacteria that can synthesize these vitamins, and how they influence the growth and proliferation of the gut microbiota and the overall intestinal health. 

## 2. Vitamin B1/Thiamin

Thiamin is an essential cofactor required for several enzymes, especially in glycolysis, the tricarboxylic acid (TCA) cycle, and the pentose phosphate pathway [23,24]. Several bacteria can synthesize free thiamine or its active form, thiamine pyrophosphate (TPP) [25]. However, bacteria belonging to the enterotype 2 class have higher thiamine biosynthetic capacity [10]. This class of bacteria, mainly Prevotella and Desulfovibrio, is overexpressed with four enzymes—hydroxymethylpyridine kinase, phosphomethylpyridine kinase, thiamine-phosphate pyrophosphorylase, and thiamine-monophosphate kinase—crucial for thiamin biosynthesis [10]. Additionally, more than 90% of other gut-associated Bacteroides possess the ability for thiamin biosynthesis and the thiamin transporter genes [26]. Dietary free thiamine is absorbed through carrier-mediated transportation such as high-affinity thiamin transporter 1 (THTR-1) and 2 (THTR-2) located in the epithelium and mucosa of the small intestine [23,27]. If the dietary thiamine is in the bound form, such as TPP, they have to be first converted to free thiamin before it is absorbed. Likewise, the above transporters, i.e., THTR-1 and THTR_2, are primarily responsible for the absorption of bacterially produced free thiamin in the colon [25]. However, TPP produced by gut microbiota is absorbed directly by TPP transporters such as TPPT -1 [28], indicating a difference in dietary and bacterially produced TPP absorption. Although the role of thiamine on intestinal integrity is not well understood, some have suggested having certain gut-related immune regulatory functions through a recent concept involving energy metabolism. Mathis and Shoelson suggested that thiamine directs the energy balance between glycolysis and TCA cycle activities, controlling immunometabolism [29]. It is also likely to have some roles associated with intestinal-linked immune cells. It has been shown that thiamine deficiency reduces the abundance of the Payer’s patches and decreases the size of B-cell follicles, leading to a reduction in naïve B cells in female Balb/c mice [30]. Their findings indicated a potential gut-related role of thiamine in immunometabolism.

## 3. Vitamin B2/Riboflavin

Riboflavin is another micronutrient involved in the energy-producing reactions of carbohydrate, fat, and protein metabolisms [24]. Riboflavin is converted to its active forms, flavin mononucleotide (FMN) and flavin adenine dinucleotide (FAD), by flavokinase and FAD synthetase, respectively. Human gut microbiota can produce riboflavin, primarily in the large intestine [25]. Dietary riboflavin ingested in FAD or FMN forms must be converted into free riboflavin before absorption [31,32]. In humans, riboflavin absorption occurs mainly in the proximal small intestine through active carrier-mediated transportation [33]. A complete riboflavin operon is present in all Baceteroidetes and Fusobacteria, as well as in 92% of Proteobacteria [34], indicating they are the primary riboflavin producers in the gut. In addition, half of the Firmicutes are predicted to be riboflavin producers [34]. Lactic acid bacteria from dairy products also possess riboflavin biosynthesis capacity [35]. These bacteria synthesize riboflavin by utilizing guanosin-5’-triphosphate (GTP), a compound derived from the purine biosynthesis pathway, and ribulose-5-phosphate, an intermediate from the pentose phosphate pathway [36]. 

Interestingly, riboflavin is also required for the postnatal development of the gastrointestinal tract. Its deficiency was associated with crypt hypertrophy, interruptions in crypt bifurcation in rats [37,38], and loss of proliferative potentials in intestinal cells [39]. These changes were seen in the postnatal and post-weaning stages [38]. The changes were irreversible even after the repletion of riboflavin in both in vivo [40] and in vitro experiments [39]. Williams and colleagues have shown that riboflavin deficiency reduced villus number but increased its length [41,42]. Riboflavin depletion in humans was also associated with shorter duodenal crypt and low cell division [43]. In vitro studies using Caco-2, HCT116, and HT29 cells demonstrated potential mechanisms of riboflavin deficiency phenotype. They include inhibiting cell growth by reducing cellular ATP generation and elevating oxidative stress [39], defective mitosis, and accumulation of aneuploidy cells [43]. The changes in this intestinal morphology might also be related to its adaptive responses to deficiency-related stresses [42]. On the other hand, riboflavin supplementation increased the abundance of bacteria that cannot synthesize riboflavin, such as *Faecalibacterium prausnitzii* and *Roseburia* spp. [33]. Higher intake was also associated with an increase in the abundance of *Prevotella* spp. but Bacteroides’ concentration was decreased in lactating women [44].

## 4. Vitamin B3/Niacin

Vitamin B3, known as nicotinamide, nicotinic acid, or niacin, is converted into its active form, nicotinamide adenine dinucleotide (NAD), which is essential for many critical metabolic processes, primarily as a redox cofactor [45]. Similar to other higher organisms, intestinal bacteria synthesize vitamin B3 mainly from amino acid tryptophan but using a unique pathway [46,47]. According to the genetic assessment, the Bacilli class contains only 4, the Clostridia class contains 44, and Proteobacteria contains 29 potential niacin synthesizing bacteria [34]. *Bacteroides fragilis*, *Prevotella copri*, and *Ruminococcus lactaris* can also produce vitamin B3 in the gut as they possess a vitamin B3 biosynthesis pathway [34,48]. Moreover, niacin-responsive transcription factor NiaR (YrxA) is present in a diverse group of Bacillus and Clostridium bacteria, meaning they can undergo de novo synthesis of NAD [49,50]. The human and mouse colonic epithelial cells possess an efficient, specific, and regulated mechanism for the uptake of vitamin B3. The bacterially synthesized vitamin B3 contributes to local colonocyte nutrition and maintains the morphology of intestinal stem cells [51].

A study from the FoCus cohort identified links between vitamin B3 and intestinal microbial composition [52]. They found a significant association between vitamin B3 deficiency and low α-diversity and the abundance of Bacteroidetes in obese individuals [52]. The abundance of this bacteria was significantly higher during a gut-targeted delayed-release of nicotinic acid but not the nicotinamide in those obese subjects [52]. Qi and colleagues isolated intestinal crypt cells from a C3H/HeN conventionally raised mouse and treated them with vitamin B3. Vitamin B3 treatment with 1200 ug/mL significantly increased the growth rate of organoids [45]. Vitamin B3 plays a vital role in reducing inflammation and causes relapsing inflammatory bowel diseases such as ulcerative colitis when deficient. It controls inflammation by inhibiting vascular permeability in intestinal tissues by activating the PGD2/DP1 signal in endothelial cells [53]. It also modulated the inflammatory response by enhancing the rate of ATP generation in Caco-2 cells [54]. Interestingly, vitamin B3 engages in various metabolic reactions that alter the cellular redox state and rapamycin signalling pathway [51], thus suppressing the colon’s inflammations [53].

Furthermore, vitamin B3 protects colonic epithelial cells against the dextran-sulfate-sodium (DSS)-induced apoptosis and promotes cell proliferation in mice. It maintains the intestinal epithelium barrier by activating the D prostanoid 1 (DP1) receptor in macrophages and endothelial and colonic epithelial cells [53]. Lower plasma niacin levels have been observed in patients with Crohn’s disease [55]. Interestingly, retention enema containing vitamin B3 effectively promoted mucosal healing in patients with ulcerative colitis, most likely due to the downregulation of colonic inflammatory cytokines and suppression of proinflammatory gene expression [53]. In another in vitro study, cellular metabolites such as glutamine, isoleucine, ornithine, and glycerophosphocholine were downregulated, and glutamic acid was upregulated in inflamed Caco-2 cells. The impairments in the metabolite profile were ameliorated with the addition of vitamin B3 [54], which means that the therapeutic properties of vitamin B3 might be related to improving specific cellular metabolites that were impaired during acute inflammations. 

## 5. Vitamin B5/Pantothenic Acid

Vitamin B5, or pantothenic acid, is an essential precursor of coenzyme A (CoA) and acts as an acyl-carrier protein [56]. It is involved in various metabolic pathways such as the citric acid cycle, cell growth, neurotransmitter synthesis, and fatty acid oxidation [1,57,58]. Various bacteria, including *Escherichia coli*, *Salmonella typhimurium*, and *Corynebacterium glutamicum*, can synthesize vitamin B5. They use aspartate and intermediate metabolites of valine biosynthesis to produce vitamin B5 [59,60,61]. For instance, S. *Typhimurium* produces pantothenate from α-ketoisovalerate using acetohydroxy acid synthase isozyme I and dihydroxy acid dehydratase enzymes [62]. Moreover, other bacteria, such as *Lactobacillus helveticus,* require pantothenic acid for their fatty acid and biotin metabolism [63]. Dietary pantothenic acid supplementation also influences gut microbial profile. Enhanced pantothenic acid intake increased the relative abundance of Prevotella and Actinobacteria and decreased the abundance of Bacteroides in lactating women [44]. 

## 6. Vitamin B6/Pyridoxine

Vitamin B6 has six vitamers, namely, pyridoxine (PN); pyridoxal (PL); pyridoxamine (PM); and their phosphorylated forms, i.e., pyridoxal phosphate (PLP), pyridoxine phosphate (PNP), and pyridoxamine phosphate (PMP) [64]. PLP is the enzymatically most active form of vitamin B6. Vitamin B6 acts as a cofactor for many biochemical reactions, primarily involved in amino acid biosynthesis and catabolism. Besides this, it is involved in fatty acid and neurotransmitter biosynthesis and also acts as an antioxidant [65,66,67,68]. In the mammalian gut, bacteria synthesize vitamin B6 through de novo or salvage pathways. Microbes such as *Bacteroides fragilis* and *Prevotella copri* (Bacteroidetes), *Bifidobacterium longum* and *Collinsella aerofaciens* (Actinobacteria), and *Helicobacter pylori* (Proteobacteria) can produce vitamin B6 as they have these biosynthetic mechanisms [69].

Most dietary vitamins are absorbed in the small intestine; however, uptake of a certain amount of dietary and bacterially synthesized vitamin B6 still occurs in the large intestine [65] because many vitamin B6 transporters are also expressed in the mammalian colon [22]. Vitamin B6 auxotrophic prokaryotes and single-cell eukaryotes rely on importing this vitamin from their surroundings, while multicellular organisms transport it to different host organs after absorption [65]. Vitamin B6 produced in the gut is not sufficient for the host’s daily requirements. Its deficiency reduced microbial β-diversity and significantly altered intestinal metabolite compared to the control groups in rats [70]. An abundance of Lachnospiraceae_NK4A136_group [70] and Prevotella [44] were elevated with vitamin B6 deficiency, while the abundance of Bacteroides was decreased when the vitamin B6 intake was high [44]. Moreover, Bifidobacterium, Slackia, Enterococcus, Thiococcus, Klebsiella, Serratia, and Enterobacter abundances were also decreased with vitamin B6 supplementation in lactose-intolerant patients [71].

A cross-sectional study has shown an association of severity of irritable bowel disease symptoms with low dietary vitamin B6 intake [72]. A plausible explanation includes the trigger of inflammation by shifting the balance between anti-inflammatory to proinflammatory cytokines with low vitamin B6 [72]. The presence of a P2X receptor antagonist such as pyridoxal phosphate 6-azophenyl-2,4-disulfonic acid, a derivative of vitamin B6 [72], and impairment in microbiota-related intestinal metabolites such as short-chain fatty acids [70] might play a significant role in triggering inflammation. Recently, Yin and colleagues demonstrated that dietary supplementation of vitamin B6 downregulated the inflammatory cytokines and upregulated the mRNA expression of amino acid transporters in the jejunum of weaned piglets [73]. Moreover, vitamin B6 deprivation studies on aquatic animals have shown a significant decrease in the number of mucous-secreting cells, a critical factor in maintaining gut health [74]. Nevertheless, vitamin B6 deficiency did not alter the basic morphological features of enterocytes, such as cell viability, cell volume, membrane permeability, and protein content in rats, but decreased calcium transport flux [75]. 

## 7. Vitamin B7/Biotin

Vitamin B7 acts as a coenzyme for several biochemical reactions, such as glycolysis [76] and cell signalling and epigenetic regulations [77,78]. It also controls gene expression, including nuclear factor *kappa B* (*NF*-*κB*), through histone binding mechanism, commonly known as biotinylation [79,80]. Therefore, this vitamin may also have anti-inflammatory effects [81,82]. Biotin is primarily synthesized from either malonyl CoA or pimeloyl-CoA [83,84]. Enzymes of the biotin biosynthesis pathway are overrepresented in enterotype 1, enriched in Bacteroides [10]. Bacteria that can produce vitamin B7 include *Bacteroides fragilis*, *Prevotella copri*, *Fusobacterium varium*, and *Campylobacter coli* [34]. In contrast, others are extensive vitamin B7 reducers, such as *Lactobacillus murinus* [85].

## 8. Vitamin B9/Folate

Folate, also known as vitamin B9, is a conjugate form of 4-aminobenzoic acid and L-glutamic acid. Vitamin B9 is supplied to the host primarily through diet and partially by gut microbiota [86]. Folate is an essential methyl donor nutrient that provides one-carbon units. It is also involved in synthesizing S-adenosylmethionine (SAM) required for cellular biosynthesis and DNA methylation [87]. This vitamin is vital for replicating and restoring nucleic acids, thus affecting cell survival rate and proliferation when deficient [88]. Additionally, folate is involved in regulating gene activities, regenerating the lining of the intestine, producing necessary chemicals for proper brain function, decreasing the growth of lymphocytes, and reducing natural killer cell cytotoxicity [89,90,91]. Thus, every living cell requires folate to perform a variety of these biochemical and biosynthetic processes. These cellular reactions are universal, but their metabolic pathways differ from organism to organism. Organisms such as fungi, plants, bacteria, and some specific archaea can undergo folate biosynthesis, and they use a similar pathway with slight modifications [92,93,94,95,96]. There are several bacteria that can produce folate in the gut, which include *Bacteroides fragilis*, *Prevotella copri*, *Clostridium difficile*, *Lactobacillus Plantarum*, *L. reuteri*, *L. delbrueckii* ssp. *bulgaricus*, *Streptococcus thermophilus*, *Bifidobacterium* spp. (some species), *Fusobacterium varium*, and *Salmonella enterica* [34,97]. Out of these bacteria, Bifidobacterium species are well studied. They are categorized on the basis of their folate-producing ability: high folate producers—*Bifidobacterium bifidum* and *B. longum* subsp. *Infantis*, and low folate producers—*B. breve*, *B. longum* subsp. *longum*, and *B. adolescentis*. Synthesis of folate requires one pterin moiety originating from 6-hydroxymethyl-7,8-dihydropterin pyrophosphate (DHPPP) and a para-aminobenzoic acid (pABA). The latter is an intermediate formed by cleaving pyruvate with 4-amino-4-deoxychorismate lyase enzyme [98]. Such enzymes are mostly confined to the genomes of Bifidobacterium species, including *B. adolescentis* and *B. dentium* Bd1 [99,100]; thus, they produce folate when DHPPP is available. Other common folate-producing bacteria are Lactobacilli. Unlike Bifidobacterium, several Lactobacillus species, including *L. plantarum*, *L. sakei*, *L. delbrueckii*, *L. reuteri*, *L. helveticus*, and *L. fermentum* can produce DHPPP; therefore, they synthesize folate when pABA is available [98]. Nevertheless, Lactococcus and Streptococcus possess a complete pathway for the de novo folate biosynthesis and do not require a supply of either DHPPP or pABA [101]. 

Due to its crucial role in methyl donor production, folate deficiency significantly impairs DNA replication. Folate depletion causes an increase in the intestinal mucosal crypt depth in the duodenum and jejunum [102], resulting in a reduced villus to crypt ratio [103]. In methyl donor-deficient mice induced by feeding a folate-deficient diet accompanied by an antibiotic, succinylsulfathiazole (1%), also had increased crypt depth and altered intestinal cell differentiation [87]. In rats, folate deficiency causes megaloblastic changes in the epithelial cell nuclei [102] and reduced crypt mitosis [103]. These changes were more remarkable in the ileum with crypt elongation, increased goblet cells, and decreased Paneth cells [87]. Thus, folate deficiency significantly alters intestinal cell morphology and is associated with increased occurrence of intestinal carcinogenesis [104,105]. Concomitant depletion of folate, riboflavin, vitamin B-6, and vitamin B-12 alter Wnt- signalling in the mouse colon and decrease apoptosis in the epithelium cell [106]. Unexpectedly, these changes are irreversible, even with the repletion of folate [103]. Although the gut bacteria can produce some folate, a folate-deficient diet significantly alters microbial diversity in the mice. It is shown that the abundances of Bacteroidales and Clostridiales decreased, and abundances of Lactobacillales and Erysipelotrichaceae taxa increased in folate-deficient mice [87].

## 9. Vitamin B12/Cobalamin

Vitamin B12, also known as cobalamin, is one of the largest and most complex vitamins [107]. The other forms of this vitamin include cyano-, methyl-, deoxyadenosyl-, and hydroxy-cobalamin. The cyano form is found as traces in diet, and it is used as a dietary supplement [108]. Like folate, cobalamin is involved in methyl donor synthesis, such as SAM. These methyl donors are crucial for nucleic acid synthesis and protein and lipid metabolism [109,110]. It is used as a cofactor for methionine synthase in sulphur amino acid metabolism to recycle homocysteine to methionine [111,112]. Cobalamin is also vital for the proper functioning of the central nervous system and the synthesis of red blood cells [110,111,113]. 

There are limited bacteria that can synthesize vitamin B12 in the human gut, and most of them use precorrin-2 as a precursor [34]. Approximately 20% of gut bacteria can produce vitamin B12, and more than 80% of gut bacteria require B12 for their metabolic reactions [114,115]. These include *Pseudomonas denitrificans*, *Bacillus megaterium*, and *Propionibacterium freudenreichi*, *Bacteroides fragilis*, *Prevotella copri*, *Clostridium difficile*, *Faecalibacterium prausnitzii*, *Ruminococcus lactaris*, *Bifidobacterium animalis*, *B.infantis*, *B.longum*, and *Fusobacterium varium* [34,48,116,117,118,119,120]. Notably, the first three bacteria are commercially used for vitamin B12 production [121]. Biosynthesis of B12 by microorganisms involves almost 30 genes and uses either aerobic or anaerobic pathways. The aerobic pathway has been studied in *Pseudomonas denitrificans,* and the anaerobic pathway has been studied in *Salmonella typhimurium*, *Bacillus megaterium*, and *P. shermanii* [122]. In general, *Lactobacillus* spp. were thought not to have a vitamin B12 biosynthetic pathway. However, the discovery of the conversion of glycerol into propanediol in lactic acid bacteria demonstrated their ability to produce vitamin B12 [121]. 

**Figure 1:**
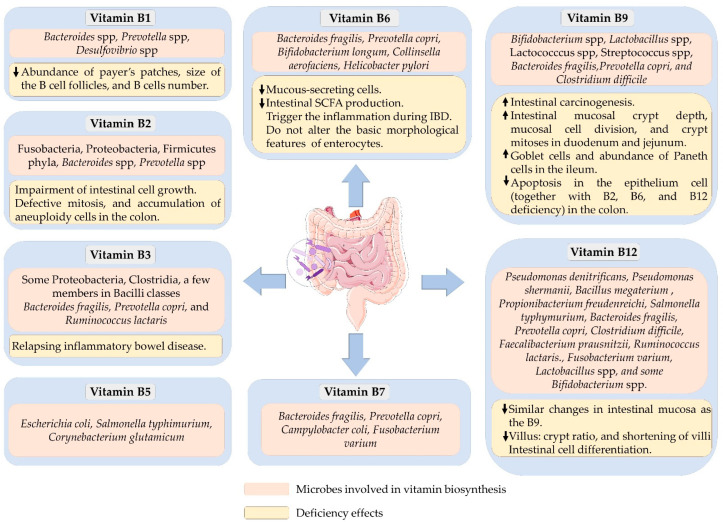
A schematic illustration listing the bacteria that can synthesize B vitamins and the effects of B vitamin deficiencies on gut health.

On the other hand, several other bacteria, including Bacteroides, do not have a vitamin B12 biosynthesis capability. However, most of them possess vitamin-B12-dependent enzymes [114]. The optimum functioning of these enzymes depends on dietary supply. The effects of vitamin B12 deficiency on colon morphology are similar to that of folate deficiency as they are closely associated with several cellular metabolic reactions. However, the impact of its deficiency on colon inflammation is not conclusive. Benight and colleagues reported that vitamin B12 deficiency protects against DSS-induced inflammations in C57BL/6 mice [123]. Contrarily, others demonstrated a reduction in cell differentiation and intestinal barrier in vitamin-B12-deficient rats [124]. Moreover, in patients with a vitamin B12 deficiency, the villus becomes shorter with a reduced villus/crypt ratio than in the control group [125]. Similarly, a dietary deficiency or surplus of vitamin B12 may likely influence the growth of gut microbiota. Unexpectedly, vitamin B12 deficiency did not alter gut microbial composition in healthy mice but altered it in DSS-induced colitis mice [126]. The short time (28 days) applied to induce the deficiency might be one of the reasons that gut microbial composition was not affected in the healthy mice. It was also likely that the animals that practice coprophagy could have maintained their vitamin status by eating feces [127]. However, the gut microbial profile in humans is influenced by the host vitamin B12 status. Vitamin B12 supplementation in humans increased the relative abundance of Prevotella, but decreased the abundance of Bacteroides [44]. Likewise, the relative abundance of Bacteroides has reduced with vitamin B12 supplementation in C57BL/6 mice [115]. Lurz and colleagues also showed that vitamin B12 supplementation in mice significantly decreased Parabacteroides and Lactobacillus and increased *E. coli* and Enterococcus abundances in a murine model of colitis [126]. Figure 1 summarizes the key intestinal bacteria that can produce B vitamins and their deficiency effects on gut health.

## 10. Factors Affecting Gut Microbial B-Vitamin Synthesis

The gut microbial vitamin B synthesis is affected by various factors, including antibiotics, free radicals, diet, and even an individual’s genetic make-up. The responses of exposure to antibiotics in B-vitamin synthesis are varied based on the type of antibiotics used. For instance, adding penicillin and aureomycin to the diet increased hepatic vitamin B2 concentration and excretion of B2 and B3 in the urine in male rats [128]. However, the administration of streptomycin [129] and cycloheximide [129] reduced vitamin B9 and B12 concentrations in the liver. The mixed responses of the vitamin synthesis upon antibiotic exposure are not well understood, but they might likely be caused by the selective alterations of the intestinal microbiota.

On the other hand, free radicals are chemical species that contain an unpaired electron and can induce oxidative stress [130]. One such example is nitric oxide, which forms complexes with metal ions, including cobalt [131], a structural component of vitamin B12, and thus makes it unavailable for bacterial vitamin B12 biosynthesis. Moreover, exposure of vitamin producers, such as *B. fragilis*, to free radicals such as hydrogen peroxide can suppress their growth [132], thereby reducing vitamin biosynthetic capacity. 

Lastly, the host genetics, dietary habits, and lifestyle also shape the gut microbial profile [133,134]. Variants in human genes are associated with gut architecture and microbiome composition [133]. The presence of distinct vitamin B biosynthetic pathways in the human gut microbiota supports the notion that human genetic variation affects vitamin B synthesis [34]. Interestingly, Bonder and his colleagues identified a single nucleotide polymorphism that was associated with the abundance of *Bifidobacterium genus* [135], bacteria that produces several B vitamins. Moreover, a host diet acts as the substrates for those bacteria living in the gut and its impacts on gut microbial profile has been extensively studied [134,136,137]. Diets containing prebiotics and other dietary nutrients such as micronutrients and polyphenols can significantly influence the growth of beneficial bacteria [138,139], including vitamin producers. Some vitamins, such as riboflavin, act as a redox mediator and stimulate the growth of auxotrophic bacteria such as *Faecaibacterium prauznitsii* [135]. On the contrary, limiting those substrates will increase the competition between the microbes and microbes and the host in an environment where a symbiotic relationship is imminent [34]. 

## 11. Conclusions

B vitamins act as cofactors for several cellular metabolic reactions. These vitamins are usually supplied primarily through the dietary intake to meet the host’s daily requirements, including nourishing the intestinal ecology. Our gut microbiota can produce a certain amount of B vitamins. The biosynthesis of these vitamins in the gut is influenced by several factors, including exposure to antibiotics and free radicals, genetic make-up, dietary habits, and lifestyle. The bacterially synthesized vitamins are not enough to supply daily requirements for the host and gut microbiota. Competitions between the gut microbes and the host and microbes to microbes create the risk of vitamin shortage in the intestine if dietary supply is not optimum. Their status can affect the gut microbial composition, colonic health, and overall host metabolism. The gut microbiota of individuals are highly diverse; therefore, biosynthesis of B vitamins and their requirements are also different from person to person. The roles of microbially produced metabolites, including B vitamins, in intestinal health and regulating the host’s cell signalling continue to be discovered. Thus, opportunities exist to investigate how dietary B vitamins affect gut–host interactions.
